# Investigating the Size-Dependent Binding of Pristine nC_60_ to Bovine Serum Albumin by Multi-Spectroscopic Techniques

**DOI:** 10.3390/ma14020298

**Published:** 2021-01-08

**Authors:** Shufang Liu, Shu’e Wang, Zhanzuo Liu

**Affiliations:** School of Public Health, Cheeloo College of Medicine, Shandong University, Wenhuaxi Road 44, Jinan 250012, China; wse@sdu.edu.cn (S.W.); liuzhanzuo2021@163.com (Z.L.)

**Keywords:** nC_60_, fullerene, bovine serum albumin, inner filter effect, fluorescence

## Abstract

The morphology of nanomaterials may affect their interaction with biomacromolecules such as proteins. Previous work has studied the size-dependent binding of pristine nC_60_ to bovine/human serum albumin using the fluorometric method and found that the fluorescence inner filter effect might affect this interaction. However, if it is necessary to accurately calculate and obtain binding information, the fluorescence inner filter effect should not be ignored. This work aimed to further investigate the effect of the fluorescence inner filter on the interaction between pristine nC_60_ with different particle sizes (140–160, 120–140, 90–110, 50–70, and 30–50 nm) and bovine serum albumin for a more accurate comprehension of the binding of pristine nC_60_ to bovine serum albumin. The nC_60_ nanoparticles with different size distributions used in the experiments were obtained by the solvent displacement and centrifugation method. UV-Vis spectroscopy and fluorescence spectroscopy were used to study the binding of nC_60_ with different size distributions to bovine serum albumin (BSA) before and after eliminating the fluorescence inner filter effect. The results showed that the fluorescence inner filter effect had an influence on the interaction between nC_60_ and proteins to some extent, and still did not change the rule of the size-dependent binding of nC_60_ nanoparticles to BSA. Further studies on the binding parameters (binding constants and the number of binding sites) between them were performed, and the effect of the binding on BSA structures and conformation were also speculated.

## 1. Introduction

As a kind of classic carbon nanomaterial, due to their unique structural characteristics and physical and chemical properties, nC_60_ nanomaterials and their derivatives have great application potential in many fields, such as chemistry, life sciences, materials sciences, and biomedicine. [1,2,3]. Therefore, research on their safety and biological effects has also attracted great attention, in which the interaction between nC_60_ (and its derivatives, most of which are water-soluble, such as fullerol, carboxylic acid, and amine) and proteins is an important part of their biological effects, and detailed comprehension of their interactions with proteins is fundamentally important for their future biomedical applications.

Previous studies have shown that nC_60_ and its derivatives can bind to a variety of proteins (peptides) and change their conformation and structure, and the morphological characteristics of nC_60_ and its derivatives have a certain effect on these bindings [4,5,6,7,8,9,10,11,12]. Since unmodified nC_60_ is not soluble in water, most of the studies were on water-soluble nC_60_ derivatives. However, due to the limitation of experimental methods, the types and quantity of modification groups of nC_60_ derivatives obtained by different research groups are different, which leads to the lack of comparability of data among different research groups. Even for the study of the interaction between pristine nC_60_ and proteins, due to the different preparation methods of nC_60_ nanoparticles, the morphology characteristics (morphology, size, particle size distribution, surface area, etc.) of nC_60_ aqueous dispersion obtained by different research groups are also different, so the research results of different research groups are not completely consistent. 

In our previous work, in order to eliminate other factors of the interaction, we obtained nC_60_ with different particle sizes by the centrifugation method, and confirmed the size-dependent binding of pristine nC_60_ to human serum albumin (HSA)/bovine serum albumin (BSA) by fluorescence quenching spectroscopy [13]. However, the binding details and mechanism need to be further discussed.

To quantify the binding of small molecules/nanoparticles to proteins, several homogeneous methods have been developed, such as isothermal titration calorimetry (ITC) [14], fluorescence quenching or enhancement [15], fluorescence polarization (FP) [16], and Förster resonance energy transfer (FRET) [17], among which the fluorescence method has been widely used because of its high sensitivity and selectivity, simplicity, and rapidity. As an important and effective method of fluorescence analysis, fluorescence quenching plays a prominent role in studying the interaction between biological macromolecules and coexisting substances. However, when the fluorescent molecules interact with coexisting substances such as small molecules/nanoparticles, the fluorescence inner filter effect is often neglected, and thus a rough or even incorrect conclusion may be drawn. 

If there are small molecules or other substances in the solution that can absorb the excitation light (primary inner filter effect) or the emission light of fluorescence substances (secondary inner filter effect), the fluorescence is weakened, which is called the inner filter effect (IFE) [14,18]. The IFE was previously considered an error in fluorescence measurement, but due to its simple operation and high sensitivity, as well as the lack of a need to modify the donor or connect the donor with the receptor, it has been widely used in the detection of enzyme activity, pesticides, metabolites, small molecule chemicals, etc. [19,20,21]. 

In our previous work, we found that particle size has an obvious effect on the binding of nC_60_ to HSA and BSA. nC_60_ nanoparticles with smaller size distribution have stronger binding to HSA/BSA proteins. At a lower concentration, considering the low absorption intensity of nC_60_ in the UV-Vis region, the existence of the inner filter effect does not change the size-dependent binding of nC_60_ to proteins. However, if we need to further clarify the law of the nanoparticle size on the interaction between nC_60_ and proteins, and then judge the mechanism of the interaction, the inner filter effect should be involved and deducted in the calculation.

Based on the research background of the interaction between nC_60_ (and its derivatives) and proteins, to further understand the influence of particle size on the interaction between nC_60_ and proteins, and to obtain the mechanism of the interaction, we further studied the inner filter effect on the interaction between nC_60_ and several proteins, and discussed the possible mechanism of said interaction. On the basis of the obtained binding information, the effect of the binding on BSA structures and conformation were also speculated.

## 2. Experimental Methods

### 2.1. Materials

C_60_ powder (purity 99.9%) was purchased from Henan Puyang Yongxin Fullerene Technology Co. Ltd. (Puyang, China). BSA, HSA (free fatty acid fraction V), and phosphate buffer saline (PBS) premixed powder (pH = 7.2–7.4) were obtained from Sigma-Aldrich ((St Louis, MO, USA). During the experiments, the nC_60_ dispersions were kept away from light. Distilled ultra-pure water (18.3 MΩ) was obtained from the Milli-Q plus system (Millipore, Bedford, MA, USA) and used in all experiments. All of the experiments were performed at room temperature.

### 2.2. Instruments

A UV-2450 spectrophotometer (Shimadzu, Tokyo, Japan), HT-7700 transmission electron microscopy (TEM) (Hitachi, Tokyo, Japan), and a FL-4500 spectrofluorometer equipped with an Xe lamp as a excitation light source (Hitachi, Japan) were used.

### 2.3. Preparation and Characterization of nC_60_ Dispersions

Stock aqueous dispersion of nC_60_ nanoparticles and nC_60_ dispersions with different particle size distributions were prepared by the centrifugal method according to our previously published work [13]. According to the different centrifugal velocities, 5 nC_60_ dispersions with different particle sizes were finally obtained and named S_4b_, S_0_, S_4_, S_8_, and S_12_, and the average size distributions of 5 dispersions was in the order of S_4b_ > S_0_ > S_4_ > S_8_ > S_12_ (S_4b_: 140–160 nm, S_0_: 120–140 nm, S_4_: 90–110 nm, S_8_: 50–70 nm, and S_12_: 30–50 nm) [13].

### 2.4. Fluorescence Spectra Measurements of Proteins

A protein stock solution of 0.5 mL (1.0 × 10^−4^ mol/L) and an nC_60_ dispersion of different volumes was added to 5 colorimetric tubes with a volume of 10 mL in order to obtain a series of concentrations of nC_60_–protein mixture dispersions, then the volume was fixed to 5 mL with PBS buffer, and the 5 mixtures were thoroughly mixed.

The fluorescence spectra measurements of the above 5 mixtures were performed on an FL-4500 spectrofluorometer equipped with a 1.0 cm quartz cell (laser source: Xe lamp; λex = 280 nm; excitation and emission slit width: 5 nm; scan speed: 1200 nm/min; photomultiplier tube (PMT) voltage: 700 V), and the intensity values at the maximum fluorescence emission wavelength of BSA were recorded. All of the measurements were done at room temperature.

### 2.5. Determination of Absorbance of nC_60_–Protein Systems

The determination of the UV-Vis absorbance of the 5 mixtures at 280 nm and 330 nm in the experiments were performed on a UV-2450 spectrophotometer equipped with a 1.0 cm quartz cell (scan speed: medium). All of the measurements were done at room temperature.

### 2.6. Mathematical Correction

For protein–nC_60_ systems, the following mathematical correction formula was used to correct the fluorescence data obtained [22]:

(1) $$F_{corr}=F_{obs}\times e^{(A_{ex}+A_{em})/2}$$

In the formula, *F_obs_* is the maximum fluorescence intensity without internal filtering effect correction; *F_corr_* is the maximum fluorescence intensity after internal filtering effect correction; *A_ex_* is the absorption value of the mixed solution at the excitation wavelength (λex = 280 nm); *A_em_* is the absorption value of the mixed solution at the emission wavelength (λem = 340 nm).

### 2.7. Determination of the Surface Hydrophobicity Index (SHI) of BSA

A buffer of 5 mL of 0.04 mol/L PBS and 0.10, 0.25, 0.50, 1.00, or 2.00 mL of BSA were added to 10 mL colorimetric tubes. An appropriate amount of nC_60_ with different particle sizes was added to each tube, and the volume was fixed with distilled water to 5 mL; the final concentration of nC_60_ with different particle sizes in each tube was fixed at 9.68 × 10^−6^ mol/L (relatively high concentration). The mixture was mixed well and reacted in the dark for 5 min at room temperature. The excitation wavelength was set at 380 nm, and the fluorescence emission spectrum (420–550 nm) was determined by scanning the above solution. In the presence of an excessive fluorescent probe (8-anilino-1-naphthalene sulfonate (ANS)), the slope of the straight line obtained by plotting the fluorescence intensity at 470 nm against the BSA concentration was the surface hydrophobicity index (SHI) of BSA.

## 3. Results and Discussion

### 3.1. The Existence of Fluorescence Inner Filter Effect in nC_60_–Protein (HSA/BSA) Systems

**Figure 1:**
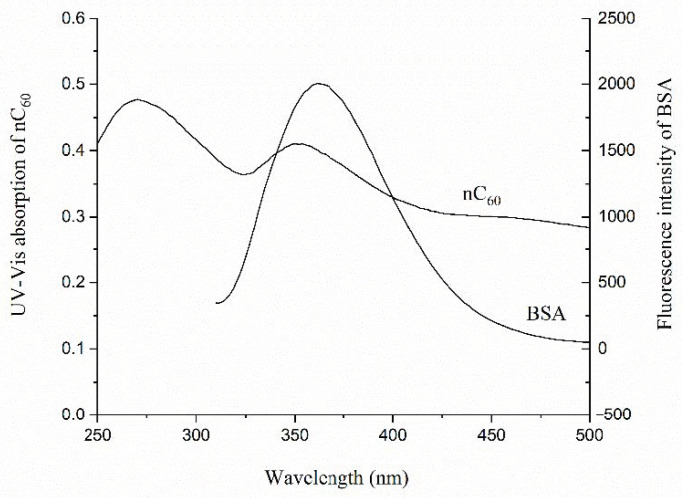
The overlap between the UV absorption spectrum of nC_60_ and the fluorescence emission spectrum of proteins (bovine serum albumin (BSA)).

Fluorescence spectroscopy is often used to study the interaction between proteins and other substances [15,23]. In previous studies, we used the fluorescence method to study the interaction between nC_60_ and several proteins. It was found that nC_60_ can quench the fluorescence of proteins (BSA and HSA) with a dose–response relationship, and the smaller the particle size of nC_60_, the stronger its ability to quench protein fluorescence [13]. However, by scanning the UV absorption spectrum of nC_60_ and the fluorescence emission spectrum of protein (BSA and HSA, λex = 280 nm), there was an overlap between them (Figure 1). This means that nC_60_ might have absorbed the excitation light and the emission light of the proteins, the so-called fluorescence internal filtering phenomenon, and thus, the fluorescence intensity of the proteins decreased. Due to the low concentration ranges and the weak absorbance of the nC_60_ nanoparticles in the experiment, the effect of the fluorescence inner filter on the size-dependent binding law of nC_60_ to the proteins was small and can be neglected [13]. However, to calculate the binding constant and the number of binding sites of the interaction between nC_60_ and the proteins, and to comprehend the mechanism of the interaction, the influence of the inner filter had to be considered and deducted. 

### 3.2. Calculation of the Fluorescence Inner Filter Effect in the System

**Figure 2:**
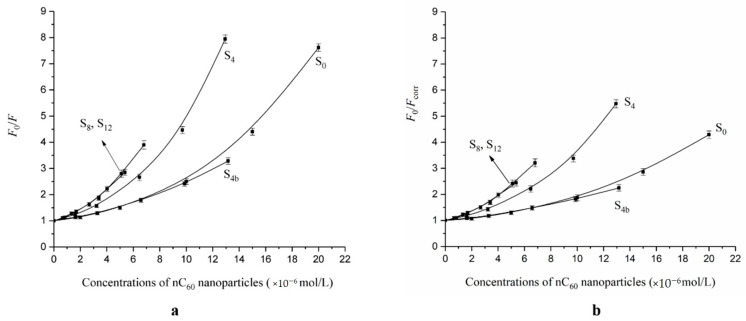
The fluorescence quenching plots of BSA by nC_60_ with (**a**) and without (**b**) correction of the fluorescence inner filter effect.

In order to comprehend the fluorescence inner filter effect on the interaction between nC_60_ with different particle sizes and BSA, we used Formula (1) for fluorescence correction, and obtained the fluorescence quenching plots of BSA by nC_60_ before correction (a) [13] and after correction (b) of the fluorescence inner filter effect (Figure 2). 

Figure 2 shows that the SternVolmer plots for the fluorescence of BSA by nC_60_ was a curve bending to the *Y*-axis before the inner filter effect was corrected. After the correction of the inner filter effect, at the same concentration of nC_60_ nanoparticles, the degree of fluorescence quenching of the proteins decreased to a certain extent, and the Stern–Volmer plots still approached curves bending to the *Y*-axis, while the size-dependent quenching effect of nC_60_ on BSA remained unchanged. 

### 3.3. The Binding Constants and Binding Sites of nC_60_ Nanoparticles to BSA

Due to the different quenching mechanisms, the fluorescence quenching mainly included dynamic quenching and static quenching. Dynamic quenching refers to the collision between the quenching agent and the excited-state fluorescence molecules during the fluorescence lifetime, which caused the fluorescence molecule to return from the excited state to the ground state in the way of non-radiative transition without emitting fluorescence, resulting in the fluorescence quenching effect of fluorescence molecule. This process can be described by the Stern–Volmer linear equation [24]:

$$F_{0}/F=1+k_{q}\tau_{0}c=1+K_{sv}c$$

In the equation, *F*_0_ and *F* represent the fluorescence intensity at the maximum emission wavelength of BSA fluorescence spectra in the absence and presence of a quenching agent, respectively; *τ*_0_ is the average fluorescence lifetime of fluorescence molecules (such as biomacromolecules) in the absence of a quenching agent, generally 10^−8^ *s*; *K_SV_* is the dynamic quenching constant (L/mol); *c* is the concentration of the added quenching agent (mol/L); *k_q_* is the dynamic quenching rate constant (L/(mol·s)), and the maximum dynamic quenching rate constant of various fluorescence quenching agents for biomacromolecule is approximately 2.0 × 10^10^ (L/(mol·s)) [12].

Assuming the fluorescence quenching mechanism of nC_60_ to BSA belongs to dynamic quenching, the Stern–Volmer plots were drawn after correction of the inner filter effect (Figure 2). The corrected Stern–Volmer plots are rising curves bending to the vertical axis, which indicate that either both dynamic quenching and static quenching occurred between nC_60_ and BSA at the same time, or single static quenching occurred [24].

For the fluorescence quenching caused by the combination of dynamic and static quenching, the retained fluorescence fraction (*F*/*F*_0_) is the product of the fraction of the uncomplexed fluorescence molecules and the fraction of the fluorescence molecules not quenched by the collision encounter:

(2) $$\frac{F}{F_{0}}=f\frac{\gamma }{\gamma +k_{q}[Q]}$$

In the equation, $\gamma =\frac{1}{\tau_{0}}$, where [*Q*] is the concentration of the quenching agent. Take the reciprocal of the above formula and rearrange it to get:

(3) $$\frac{F_{0}}{F}=\frac{1}{f}(1+k_{q}\tau_{0}[Q])$$

(4) $$\frac{F_{0}}{F}=\frac{1}{f}(1+K_{SV}[Q])$$

If static quenching occurs between the quenching agent (*Q*) and the fluorescent molecules (*M*), it is assumed that a ground-state complex is formed between the quenching agent (*Q*) and the fluorescent molecules (*M*) as *MQ_n_*, namely:

$$nQ+M↔MQ_{n}$$

The binding constant (*K*) between the quenching agent and the fluorescent molecules can be expressed as:

(5) $$K=\frac{\left[MQ_{n}\right]}{[M]{\left[Q\right]}^{n}}$$

According to the relationship between fluorescence intensity and quenching agent concentration, it can be deduced as follows:

(6) $${\left[M\right]}_{0}=[M]+[MQ_{n}]$$

(7) $$\frac{F_{0}-F}{F}=\frac{{\left[M\right]}_{0}-[M]}{\left[M\right]}=\frac{\left[MQ_{n}\right]}{\left[M\right]}=K{\left[Q\right]}^{n}$$

Rearrange Equation (6) to get Equation (7):

(8) $$\frac{F_{0}}{F}=1+K{\left[Q\right]}^{n}$$

If the quenching behavior of the quenching agent to the fluorescence molecules belongs to static quenching, the fraction of the fluorescence molecules that has not been complexed (*f*) can be expressed as follows:

(9) $$f=\frac{F}{F_{0}}=\frac{1}{1+K{\left[Q\right]}^{n}}$$

In Equation (6), ${\left[M\right]}_{0}$ is the total concentration of fluorescent molecules, [*M*] is the concentration of uncomplexed fluorescent molecules, and [*MQ_n_*] is the concentration of ground-state complexes generated by the quenching agent and fluorescent molecules.

From Equations (3) and (8), Equation (9) can be deduced:

(10) $$\frac{F_{0}}{F}=\frac{1}{f}(1+K_{SV}[Q])=(1+K{\left[Q\right]}^{n})(1+K_{SV}[Q])$$

Namely:

(11) $$(\frac{F_{0}}{F}-1)=K_{SV}[Q]+K{\left[Q\right]}^{n}+K_{SV}K{\left[Q\right]}^{n+1}$$

A curve can be obtained by drawing $\left(\frac{F_{0}}{F}-1\right)$ to [Q], and the values of *K_SV_*, *K,* and *n* can be obtained by simulating the curve with the nonlinear fitting function of Origin software.

Using the above method (the obtained fitting curves shown in Figure 2), the parameters (*K_SV_*, *K,* and *n*) of the interaction between nC_60_ with different particle sizes and BSA could be obtained (Table 1).

From Table 1, the *K_sv_* was in a range of 2.35 × 10^4^–12.1 × 10^4^ L/mol, and the *K_q_* was in a range of 1.21 × 10^12^–7.25 × 10^12^ L/(mol·s), which was much larger than the maximum diffusion collision quenching constant (2.0 × 10^10^ L/(mol·s)) of various quenchers on biomacromolecules, indicating that the fluorescence quenching mechanism of nC_60_ for BSA was mainly static quenching. The number of binding sites (*n*) ranged from 1.39 to 2.41, indicating that 1–2 C_60_ molecules might be bound to one BSA molecule.

Actually, the sizes of five nC_60_ nanoparticle dispersions were 40–160, 120–140, 90–110, 50–70, and 30–50 nm, respectively, which were much larger than the protein molecules. Therefore, it was difficult to incorporate nC_60_ into the binding pocket of BSA. According to the previously reported atomistic computer simulations of some albumin subdomains on a hydrophobic graphite surface [25], it was proposed that BSA might be absorbed on the surface of an nC_60_ aggregate, which caused the fluorescence quenching. According to the quenching synchronous fluorescence spectrometry, the binding site of nC_60_ to BSA was located near the tryptophan residues, indicating the conformation change of BSA.

From the BSA fluorescence quenching plots by nC_60_, with or without correcting the fluorescence inner filter effect, the apparent size-dependent binding of nC_60_ to BSA could be observed (Figure 2). At the same concentration of nC_60_, the smaller the particle size, the stronger the fluorescence quenching ability of BSA. However, interestingly, after deducting the fluorescence internal filtering effect, by calculating the binding parameters of BSA and nC_60_ with different particle sizes, it can be seen that this was not the case. It was actually more complicated. Table 1 shows that S_4_ had the strongest binding ability (*K*) to BSA and the largest number of binding sites (*n*), followed by S_0_, and that S_4b_ was the weakest. According to the size distribution of the nC_60_ nanoparticles characterized by TEM in our previous work [13], it can be speculated that compared with other particle sizes, nC_60_ nanoparticles of approximately 100 nm bind more easily to BSA because they are more sensitive to binding.

### 3.4. Effect of nC_60_ with Different Size Distributions on the Surface Hydrophobicity of BSA and Speculation of Their Binding Sites

**Figure 3:**
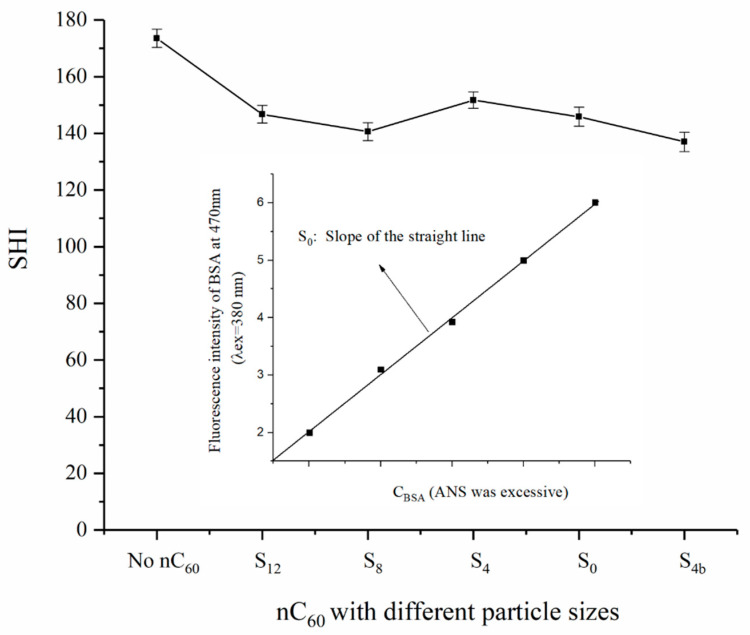
The BSA surface hydrophobicity determined using the ANS fluorescence probe method in the absence and presence of nC_60_ nanoparticles.

In order to further understand the effect of nC_60_ nanoparticles on BSA conformation and to speculate their binding sites with BSA, the effect of nC_60_ on the surface hydrophobicity of BSA was studied using the fluorescence probe method. 8-anilino-1-naphthalene sulfonate (ANS) is a common fluorescent probe, which can be non-covalently bound to the non-polar region of proteins. Its fluorescence spectrum is blue-shifted with an increase in the non-polar environment, and its fluorescence intensity also increases and shows a linear relationship with the concentration of proteins. Therefore, by determining the polarity change of the binding sites of proteins, a change of hydrophobicity and protein structure can be speculated [26,27]. In this experiment, the concentration of nC_60_ with different particle sizes was fixed at 9.68 × 10^−6^ mol/L (relatively high concentration), and their effects on the BSA surface hydrophobicity using the ANS fluorescence probe method were observed (Figure 3).

Figure 3 shows how, after adding the same concentration of nC_60_ with different particle sizes to a BSA solution, the surface hydrophobicity of BSA decreased slightly, but the effect of nC_60_ with different particle sizes on the hydrophobicity of BSA was not significant, and there were no obvious effect of nC_60_ particle size.

Previous work by synchronous fluorescence spectrometry has proved that the binding site of nC_60_ to BSA is located in the vicinity of tryptophan residues [13,28], and that the effect of nC_60_ with different particle sizes (at lower nC_60_ concentrations) on the microenvironment around tryptophan residue is also different [13]. Similarly, if the concentration of nC_60_ with different particle sizes was fixed at 9.68 × 10^−6^ mol/L, it could be seen that the influence of nC_60_ with different particle sizes on the fluorescence emission wavelength of BSA was different. The smaller the particle size of nC_60_, the more likely was a red shift of the maximum fluorescence emission wavelength of BSA, while the larger the particle size of nC_60_, the more likely was a blue shift of the maximum fluorescence emission wavelength of BSA.

Therefore, according to the experimental results of this work and our previous work, it can be speculated that the binding site of nC_60_ and BSA was closer to the position of tryptophan residues, which caused a polarity change of microenvironment around amino acid residues, and this change showed a relatively obvious size effect of nC_60_. However, the polarity change of the microenvironment around amino acid residues had little effect on the structure of BSA, and had no obvious size effect on the nC_60_ nanoparticles.

## 4. Conclusions

In this work, the size-dependent binding of nC_60_ to BSA was further calculated using the spectroscopic method. It was found that although nC_60_ with different particle sizes could quench the fluorescence of BSA with a certain particle size effect, the nC_60_ with a particle size of approximately 100 nm was easier to bind to BSA with a relatively stronger binding constant and a larger number of binding sites, which may be due to the fact that nC_60_ of this size was more suitable for its binding in the cavity around the tryptophan residues of BSA. This conclusion needs further verification, such as molecular docking, and other experimental technologies such as circular dichroism, infrared spectroscopy, Raman spectroscopy, nuclear magnetic resonance spectroscopy, microcalorimetry, laser light scattering, fluorescence polarization technology, and mass spectrometry. In addition, although nC_60_ had a certain effect on the microenvironment around tryptophan residues, it had little effect on the structure of the whole protein molecule and had no obvious particle size effect of nC_60_. Due to the characteristics of nanomaterials, many related research works have used the methods of interaction between small molecules and biomacromolecules, so there are some limitations. New research tools need to be developed. In conclusion, this work suggests that the particle size of nanomaterials, including nC_60_, has an important influence on their binding to biomacromolecules such as proteins, which provides reference for the design and future application of nanomaterials in biomedicine, such as drug carriers. 

## Figures and Tables

**Table 1:** The parameters (*K_SV_*, *K*, *n,* and *K_q_*) of the interaction between nC_60_ and BSA.

nC_60_	Binding Parameters of nC_60_ to BSA				*R*^2^ (COD)
	*K_SV_* (×10^4^ L/mol)	*K* (×10^8^ L/mol)	*n*	*K_q_* (×10^12^ L/(mol·s))	
S_12_	7.25	0.20	1.41	7.25	0.998
S_8_	5.13	1.15	1.53	5.13	0.998
S_4_	12.1	7015.84	2.41	1.21	0.999
S_0_	2.88	15.37	1.90	2.88	0.999
S_4b_	2.35	0.05	1.39	2.35	0.999

## Data Availability

All data generated or analyzed during this study were included in this article. The original datasets used or analyzed during the current study are available from the corresponding author on reasonable request.
